# Cellular Consequences of Coenzyme Q10 Deficiency in Neurodegeneration of the Retina and Brain

**DOI:** 10.3390/ijms21239299

**Published:** 2020-12-06

**Authors:** Haider Manzar, Dalia Abdulhussein, Timothy E. Yap, M. Francesca Cordeiro

**Affiliations:** 1 Imperial College Ophthalmology Research Group, Western Eye Hospital, 153-173 Marylebone Road, Marylebone, London NW1 5QH, UK; haider.manzar@southend.nhs.uk (H.M.); dalia.abdulhussein13@imperial.ac.uk (D.A.); timothyedward.yap@nhs.net (T.E.Y.); 2 Glaucoma & Retinal Neurodegeneration Research Group, Institute of Ophthalmology, University College London, London EC1V 9EL, UK

**Keywords:** coenzyme Q10, neurodegeneration, reactive oxygen species, apoptosis

## Abstract

Coenzyme Q10 (CoQ10) is a ubiquitous cofactor in the body, operating in the inner mitochondrial membrane, where it plays a vital role in the generation of adenosine triphosphate (ATP) through the electron transport chain (ETC). In addition to this, CoQ10 serves as an antioxidant, protecting the cell from oxidative stress by reactive oxygen species (ROS) as well as maintaining a proton (H^+^) gradient across lysosome membranes to facilitate the breakdown of cellular waste products. Through the process of ageing, the body becomes deficient in CoQ10, resulting in several systemic manifestations. On a cellular level, one of the consequences of CoQ10 deficiency is apoptosis, which can be visualised in tissues of the central nervous system (CNS). Diseases affecting the retina and brain such as age-related macular degeneration (AMD), glaucoma, Alzheimer’s disease (AD) and Parkinson’s disease (PD) have shown defects in cellular biochemical reactions attributed to reduced levels of CoQ10. Through further research into the pathogenesis of such conditions, the effects of CoQ10 deficiency can be counteracted through supplementation, early detection and intervention.

## 1. Introduction

Coenzyme Q10 (CoQ10) is naturally ubiquitous in the human body. It is a cofactor for mitochondrial enzymes that play a vital role in the formation of adenosine triphosphate (ATP), needed to generate energy required by the cell for its biochemical functions. CoQ10 is a lipid-soluble component of the inner mitochondrial membrane, playing a key role in the electron transport chain (ETC), where it transfers electrons generated from the reduction of fatty acids and glucose to electron acceptors. It also creates a proton (H^+^) gradient across the inner mitochondrial membrane by transporting H^+^ from the mitochondrial matrix to the intermembrane space. When H^+^ travel back along the gradient, the energy generates ATP [1,2]. Furthermore, CoQ10 also serves as a powerful antioxidant in its reduced form (CoQ10H_2_), which in turn protects the cell from oxidative stress. This counteracts the harmful effects of reactive oxygen species (ROS) that can cause damage to proteins, lipids and DNA in particular during lipid peroxidation. CoQ10H_2_ may also have indirect antioxidant effects via its potential to regenerate α-tocopherol, a form of vitamin E with antioxidant properties [2]. Finally, CoQ10 also acts as a H^+^ carrier to enable lysosomes to carry out their function in clearing cellular debris and maintaining intracellular integrity. Lysosomes require an acidic pH in order to degrade cellular debris and CoQ10 has been found to occupy lysosomal membranes, where it transports H^+^ across the membrane to maintain an acidic pH [3].

## 2. Deficiencies of CoQ10

There are three possible causes for a deficiency in CoQ10: reduced dietary intake, impaired biosynthesis or increased usage of CoQ10 by the body [2]. Endogenous supplies are generated in the liver but it can be found in organ meat, soy oil, sardines and peanuts [4]. The normal range of CoQ10 concentration in human plasma is 0.8–1.2 mg/L [5]. Ageing leads to a natural decrease in levels of CoQ10 as a result of decreased synthesis and increased degradation, and this deficit cannot be compensated for by diet [2].

Primary deficiency is associated with defects in genes directly involved in the biosynthesis of CoQ10. To date, CoQ10 and idebenone supplementation is the only treatment for deficiencies, with early detection leading to better prognosis [6]. High-dose CoQ10 supplementation of 1.2–3 g/day is typically given to adults; however, the efficacy is dependent on the nature of mutation of the biosynthetic pathway [2]. Causes of secondary deficiency include mutations of genes not directly involved in the biosynthesis of CoQ10 (APTX, ETFDH, BRAF, ANO10), or impaired CoQ10 synthesis, insufficient dietary intake and excessive cellular usage of CoQ10 [7,8].

## 3. Consequences of CoQ10 Deficiency

Normal mitochondrial function is an integral part of normal cellular function, particularly in tissues with a high metabolic activity. Higher oxygen consumption in the tissue is related to greater generation of ROS, which must be cleared. Disorders relating to CoQ10 manifest by impaired energy metabolism and protection against free radicals. This is not surprising, as the highest concentrations of CoQ10 have been observed in the tissues of the heart, kidneys, brain and muscle which is likely related to their high metabolic activity [9,10].

Although reduced ATP production and increased ROS are the main features of CoQ10 deficiency, related disorders present with great phenotypic variability. It has been found through culturing cells that *moderate* CoQ10 deficiency leads to a greater degree of ROS generation and a relatively unaffected energy synthesis but higher levels of cell death. On the contrary, *severe* CoQ10 deficiency affects ATP synthesis to a greater degree than ROS generation [11]. Alongside a known reduction in pyrimidine synthesis contributing to disease mechanisms, CoQ10 deficiency has recently been linked to impairment of the sulphide oxidation pathway. Experimental evidence has shown accumulation of hydrogen sulphide, affecting protein S-sulfhidrylation that can cause a build-up of ROS [11]. Generally speaking, primary CoQ10 deficiency is rarer than secondary deficiency [12].

In circulation, CoQ10 is carried bound to lipoproteins, in particular low-density lipoprotein (LDL) in its reduced form (CoQ10H_2_), which can be easily oxidised to CoQ10. When LDL is exposed to oxidative stress in vitro, CoQ10 is the first antioxidant to be depleted. As it is considered that CoQ10H_2_ inhibits lipid peroxidation in LDL and it has a low threshold for oxidation, the CoQ10H_2_/CoQ10 ratio can be determined through high-performance liquid chromatography and this can be used as a potential marker to determine the oxidative stress LDLs have been subjected to in vivo [13].

### Cellular Consequences of CoQ10 Deficiency

**Figure 1:**
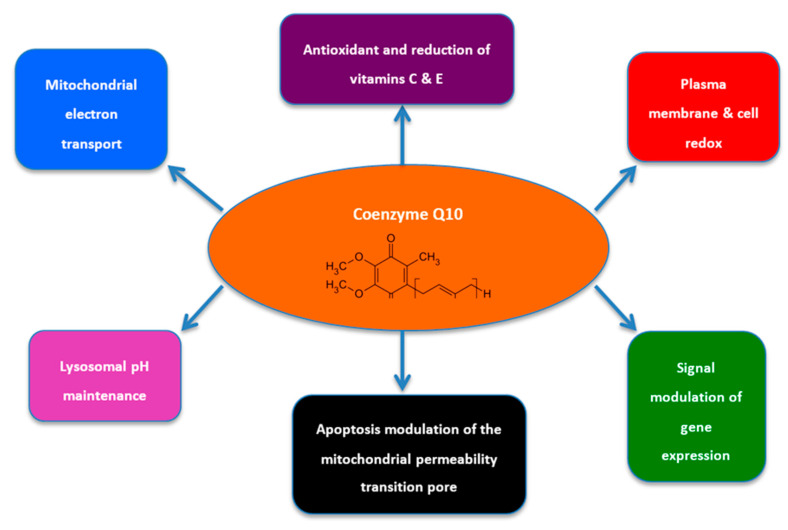
The intracellular roles of CoQ10 (adapted from De Barcelos and Haas, 2019 [16]).

**Figure 2:**
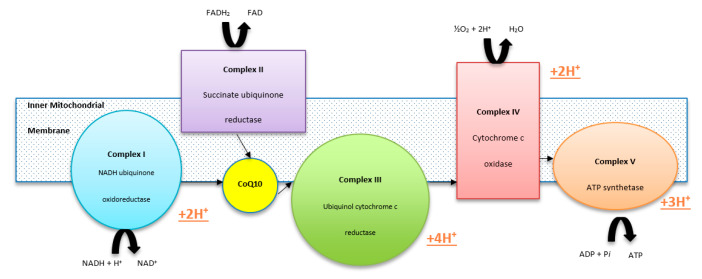
The mitochondrial electron transport chain.

CoQ10 supports cellular function through maintenance of the mitochondrial ETC, protection against free radicals and support of lysosomal function (Figure 1). Therefore, deficiencies can be traced back to their effects on cellular function. In the mitochondrial ETC, CoQ10 transports electrons from complex I (NAHD ubiquinone oxioreductase) and complex II (succinate ubiquinone reductase) to complex III (ubiquinone cytochrome c reductase) (Figure 2). The ETC is made up of three protein complexes: I, III and IV. Electrons enter the ETC from NADH via complex 1, joining a quinol in the membrane. Energy is released in the process and this is used to import 4H^+^ into the inner mitochondrial membrane. The electrons that were transferred to the quinol by complex I are now transferred to cytochrome c, which serves as a carrier. Once again, the generated energy pumps in one H^+^. The final stage involves the transfer of electrons from cytochrome c to oxygen (O_2_) by complex IV. It utilises 4H^+^ for each oxygen molecule in the process to form water (H_2_O). Complex II does not directly affect the H^+^ gradient across the inner mitochondrial membrane, but transfers electrons from succinate to quinone [14]. This flow of electrons drives oxidative phosphorylation and generation of ATP [13]. The ETC is implicated in a number of disorders, with defected functioning of specific complexes resulting in slightly different phenotypic traits. In general, CoQ10 deficiencies have been associated with reduced levels of activity in complexes II and III, and raised levels of activity in complex I [15]. 

Research has found significantly reduced levels of complex II and III activity in skeletal muscle analysis in the subjects with primary CoQ10 deficiency, along with ragged-red fibres and lipid storage defects [17]. Similarly, Gempel et al. discovered mutations in the gene coding for electron-transferring flavoprotein dehydrogenase (ETFDH) in seven patients with proximal myopathy and exercise intolerance from five separate families [18]. They were found to have raised serum creatine kinase (CK) and lactate alongside CoQ10 deficiency in skeletal muscle samples. It has been suggested that a mutated ETFDH gene leads to secondary CoQ10 deficiency and patients have shown beneficial long-term responses to CoQ10 and riboflavin supplementation [18]. The mechanism of secondary CoQ10 deficiency is thought to be a result of damage to the reducing enzyme ETFDH in fibroblasts. Since CoQ10 accepts electrons from electron-transferring flavoprotein (ETF), if the reducing enzyme is defective, a feedback loop down-regulates the synthesis of CoQ10 [18].

Lysosomes are crucial to cellular survival through recycling cellular debris and even organelles such as mitochondria. They contain over 70 enzymes involved in the digestion of cellular waste products and CoQ10 is vital in supporting their role. In deficiencies of CoQ10, the H^+^ concentration gradient required for lysosomal function is impaired. Although the impact of lysosomal deacidification is relatively under-researched, a recent in vitro study showed that a deficiency in CoQ10 is associated with a higher lysosomal pH [15]. Just like mitochondria, lysosomes possess an ETC (LETC) that uses CoQ10 to maintain a H^+^ gradient through oxidation/reduction reactions. In the LETC, electrons transfer from cytochrome b to ubiquinone, oxidising ubisemiquinone in the process, allowing H^+^ to enter across the lysosomal membrane. It therefore makes sense that a deficiency of CoQ10 will result in deacidification of lysosomes due to impaired maintenance of pH [15].

## 4. Retina

The retina is the most metabolically active tissue in the body, with the highest consumption of energy per unit area of tissue [9]. Patients with primary CoQ10 deficiency may have retinopathy as part of their syndrome, which suggests that CoQ10 may play an important role in the pathogenesis of retinal conditions. Genetic defects implicated include COQ2, PDSS1 and PDSS2 (where patients were also shown to suffer from progressive visual failure as a result of optic atrophy and cataract on top of retinopathy) [19,20]. Moreover, reduced levels of CoQ10 have been observed in tissue samples with an older age; it is unclear whether reduced levels of CoQ10 are a cause for ageing or whether it is the consequence [16]. Qu et al. found an approximately 40% lower CoQ10 concentration in younger (≥30 years old) than older (≥80 years old) human retinas [21]. A similar effect has been shown in other human tissues including the brain and heart [16,22,23,24,25]. Indeed, oxidative stress has been postulated to play a key role in the pathogenesis of many age-related diseases such as atherosclerosis, cataracts and Alzheimer’s disease [26,27,28]. The mitochondrial theory of ageing links ageing, oxidative stress and apoptosis by proposing that the accumulation of ROS with age results in greater cellular damage which mediates apoptotic mechanisms of cell death [29].

Age-related macular degeneration (AMD) is a major cause of blindness in the elderly, causing patients to suffer from a loss of central vision, which has significant impacts on quality of life. CoQ10 levels in plasma and platelets from age-matched AMD and control patients revealed lower levels in AMD patients, which suggests an association between oxidative stress (and CoQ10) and the pathogenesis of AMD [30].

The retina is exposed to more light and oxygen than most tissues in the body and also possesses a high concentration of polyunsaturated fatty acids. This leaves retinal tissue, more so at the macula, prone to oxidative stress and lipid peroxidation, respectively [30]. Furthermore, animal models have shown CoQ10 and its lipophilic derivatives such as α-tocopherol to serve as protective agents against light-induced apoptosis of retinal ganglion cells (RGCs). The result of this oxidative stress is apoptotic cell death [29]. Blasi et al. amongst others have found improved bioenergetic state of RGCs following high-dose CoQ10 supplementation (1.2–2.4 g/day) in Leber’s hereditary optic neuropathy and other age-related degenerative disorders, further highlighting its importance as a coenzyme in retinal tissue [30,31].

Glaucoma is an optic neuropathy characterized by the loss of RGC, which are key in transmitting the signal from the photoreceptors to the optic nerve, often but not exclusively associated with raised intraocular pressure (IOP). A loss of RGCs leads to progressive loss of vision in sufferers. Interestingly, animal models indicate that age-related mitochondrial defects play a central role in the pathogenesis of glaucoma [32,33,34]. Levels of CoQ10 in the human retina have been reported to decline with age [22]. The prevalence of glaucoma increases with age, hence there may be a possible increased vulnerability of RGCs in glaucomatous neurodegeneration due to a lack of CoQ10 in older age [34,35].

Intravitreal administration of CoQ10 has been seen to minimize glutamate increase in a rat model of ischaemia/reperfusion, delaying apoptosis in RGCs observed at 24 h. This has supported oxidative stress being implicated in mechanisms of RGC death, possibly via the accumulation of glutamate, with CoQ10 offering a potential neuroprotective role [32,33]. Glaucoma has also demonstrated a similar pattern of cell damage, related to glutamate accumulation, that is observed in other neurodegenerative diseases [36,37].

Animal studies have been used to demonstrate the therapeutic potential of 1600–2000 mg/kg body weight CoQ10 supplementation. In a mouse model with retinal ischaemia induced by IOP elevation, CoQ10 supplementation for 2 weeks significantly prevented the upregulation of SOD2 and heme oxygenase-1 (HO-1) [34]. Lulli et al. showed in a mouse model that CoQ10 eye drops increased RGC viability and inhibits apoptosis in response to different apoptotic stimuli such as glutamate, chemical hypoxia (Antimycin A), and serum withdrawal (FBS 0.5%), by preventing mitochondrial depolarisation [38]. In a surgically induced rat model of ocular hypertension, daily topical treatment with CoQ10/TPGS (alpha-tocopherol polyethylene glycol succinate) (0.5% *w/v* TPGS with 0.1% *w/v* CoQ10) showed significantly reduced RGC apoptosis and loss and was shown in vitro to reduce RGC vulnerability to oxidative stress induced by dimethyl sulfoxide and paraquat [39].

A prospective, randomised controlled study evaluating the effect of CoQ10 drops combined with vitamin E on patients with pseudo-exfoliative glaucoma (PEX) found that super oxide dismutase levels were significantly lower in the group treated with CoQ10 and vitamin E than in the control during the 1-month follow-up. Similar to the aforementioned rat model [39], 0.5% *w/v* TPGS was used in combination with 0.1% *w/v* CoQ10. Unfortunately, this result was not clinically correlated [40]. An on-going randomized, double-blind, controlled clinical trial in Italy is investigating the use of topical CoQ10 and vitamin E in primary open angle glaucoma (POAG), sponsored by the Italian “Instituto di Ricerche Farmacologiche Mario Negri”. Vitamin E deficiency has been linked to increased RGC apoptosis through a higher rate of lipid peroxidation [41]. This is an interesting study that intends to investigate the time to progression of POAG with topical administration of CoQ10 and vitamin E drops. On a cellular level, CoQ10 is thought to protect RGCs from micro-ischaemia induced by the suppressed release of glutamate [36].

## 5. Brain

Alzheimer’s disease (AD) and Parkinson’s disease (PD) are the most common chronic neurodegenerative diseases of the brain. Amongst others, Huntington’s disease (HD) and Friedrich’s Ataxia (FA) are neurodegenerative diseases that are implicated by deficiencies in CoQ10.

### 5.1. Alzheimer’s Disease

AD patients tend to present with short-term memory loss initially followed by overall decline in cognitive ability, often with personality changes. Histopathologically, it is defined by abnormal deposition of β-amyloid (Aβ) plaque and intracellular accumulation of neurofibrillary tangles of hyperphosphorylated tau protein. A population-based prospective cohort study of 6000 Japanese subjects demonstrated an inverse association between serum CoQ10 levels at baseline and risk of incident dementia which suggests that CoQ10 is implicated in the pathogenesis of AD and may be a predictor for its development [42]. Animal studies have also corroborated this association. Choi et al. found that supplementation with CoQ10 protected Aβ-injured neurons against Aβ-induced neurotoxicity in a concentration-dependent manner, mainly by inhibiting oxidative stress via the activation of the PI3/Akt pathway in the rat cortex. This was achieved through treating affected neurones with concentrations of CoQ10 ranging from 0 to 100 μM [43]. Yang et al. postulated that oxidative stress is enhanced in brain of transgenic mice, which promotes Aβ42 overproduction and that CoQ10 attenuated this process, and reduced Aβ accumulation [44]. A mouse model for AD treated with 2.4% CoQ10 resulted in a greater decrease in plaque area and more improved cognitive performance than those supplemented with 0.4% CoQ10. However, both concentrations yielded positive results [45]. Human studies have demonstrated that patients with AD showed far greater signs of oxidative stress than elderly controls, with increased thiobarbituric acid reactive substances (TBARS) production and nitric oxide synthase (NOS) and superoxide dismutase (SOD) activity [46]. As well as improving cognition, CoQ10 has also been shown to regulate mitochondrial function and promote ATP synthesis for further energy utilisation and cellular processes. Studies have shown CoQ10 to slow down the depletion of ATP and accumulation of lactate in mitochondria of rat AD models [47]. Unfortunately, the protective role of CoQ10 supplementation is yet to be demonstrated in humans. One potential reason for this may be the fact that once diagnosed with AD; patients are commenced on treatment immediately, making it a challenge to recruit patients for a placebo arm to compare with CoQ10. A randomized-controlled trial using 400 mg of CoQ10 supplementation three times daily for 4 months in patients with mild–moderate AD did not show a change in cerebrospinal fluid levels of Aβ and Tau [48]. The use of idebenone, a man-made product akin to CoQ10, however, has shown beneficial effects in AD patients. A large randomised control trial (RCT) administered 45 mg of idebenone two times a day to 102 AD patients over a four-month period. Results showed significantly improved cognition [49]. Another RCT found that long-term idebenone supplementation served to slow down the progression of AD as well as exhibiting improved effects on memory in patients [50]. This can be likened to the positive results seen in animal AD models of CoQ10 supplementation [47]. CoQ10 may have an important role in AD pathogenesis but its role in treatment is yet to be determined.

### 5.2. Parkinson’s Disease

PD tends to present clinically with a triad of increased rigidity, resting tremor and slowed movements, postural instability [51]. In later stages, non-motor behavioural symptoms including dementia, depression, and insomnia may arise. The key pathological process in the development of PD is death of dopaminergic cells and the presence of Lewy bodies in neurons in the substantia nigra compacta [52,53]. The exact causes of PD are not fully elucidated but genetic and environmental factors have been postulated [54,55,56]. Ultimately, there is mitochondrial dysfunction, which is triggered by oxidative stress. Proteins that have been directly linked to the disease including, PARK1/parkin, SNCA, LRRK2, PARK7/DJ-1, PINK1 are associated with mitochondria [54]. Several mechanisms have been proposed to contribute to oxidative stress and the accumulation of ROS in PD including lipid peroxidation, glutathione deficiency, and defective mitochondrial respiratory chain functions [57,58,59]. In particular, there has been increased attention to reduced complex I activity in patients with PD, both in post-mortem analysis of the substantia nigra and in platelets [57,60,61,62]. Moreover, selective inhibition of complex I in the mitochondrial electron transport chain with 1-methyl-4-phenyl-1, 2,3,6-tetrahydropyridine (MPTP) replicates the clinical and pathological features of PD in experimental models [63,64]. Current treatment strategies in PD involve the administration of levodopa with a peripheral DOPA decarboxylase inhibitor (such as selegiline) [65]. However, these do not halt the disease progress but may delay the progression of symptoms in some individuals, particularly if administered early in the course of disease. This may be due to the fact that treatment with levodopa and selegiline does not affect mitochondrial, and specifically complexes I, II/III and IV, function [66]. This finding lends further support to the theory that impaired complex I activity in PD patients is a feature of the disease rather than a consequence of the treatment. Hence, free radical mediated neuronal damage is involved in neurodegeneration, but it remains unanswered whether this is an associative or a causative relationship.

CoQ10 is a potent antioxidant and is the electron acceptor for complexes I and II [67]. Shults et al. demonstrated reduced CoQ10 levels in mitochondria from PD patients when compared to age- and sex-matched neurologically normal controls [67]. This provided an important link between CoQ10 function, mitochondrial dysfunction and PD and led to the theory that CoQ10 supplementation may be beneficial in patients with PD. Indeed, oral CoQ10 supplementation (200 mg/kg/day) in MPTP-treated mice reduced loss of dopaminergic axons compared to a standard diet [68]. Despite several studies demonstrating increased levels CoQ10, good tolerability of oral CoQ10 supplementation and improved Parkinsonism symptoms according to the Unified Parkinson Disease Rating Scale (UDPRS), such a positive effect was not observed in Phase 3 human trials with oral CoQ10 supplementation of 300, 600 or 1200 mg/day [69,70]. This may be because oral supplementation with CoQ10 has limited bioavailability at the striatum, owing partly to its large molecular weight and low aqueous solubility, which hinders its transport across the blood–brain barrier, even at high doses [31,71]. This theory is further supported by the recent findings that a PD rat model with intrastriatal CoQ10 administration, compared to no treatment, had higher dopaminergic neurons and less inflammation compared to oral administration [72].

### 5.3. Huntington’s Disease

HD manifests as gradual-onset cognitive impairment, behavioural changes and chorea. The disease is the result of a trinucleotide repeat sequence of cytosine, adenosine and guanine (CAG) [47]. Biopsies of degenerative neurons in the striatum have shown decreased levels of complex II and III activity, increased activity of complex I as well as abnormal mitochondrial morphology. The combined effect is impaired mitochondrial metabolism and hence production of ATP for cellular biochemical reactions [73]. The way in which CoQ10 is thought to manifest its effects is through disinhibiting complex II, which is usually suppressed by ROS and other toxins. This in turn lowers lactate concentrations in the striatum [47].

Several animal and human studies have shown CoQ10 to have positive prognostic effects on HD. A transgenic mouse model demonstrated prolonged survival after treatment with 400 mg/kg/day CoQ10 by delaying the onset of motor deficits [74]. Another study using magnetic resonance spectroscopy revealed elevated lactate concentrations in the striatum of HD patients, which were lowered following administration of 360 mg/day CoQ10 for two months [75]. In a larger-scale study, Kieburtz et al. in the Huntington Study Group conducted a randomised control trial on 347 patients with early signs of HD, showing that those who received 300 mg CoQ10 twice a day over a 30 month period had a slower disease progression than those receiving placebo. However, these results were not statistically significant [76]. There have been several human studies that did not show any significant improvement with administering CoQ10 in HD patients [73,76,77]. However, there is yet evidence favouring beneficial long-term effects of supplementation through its effects on intracellular concentrations of lactate, which is known to potentiate disease progression. The most important pathophysiological feature in HD is increased levels of lactate in the brain [75]. CoQ10 has been proven to lower these concentrations clinically. Administering low-dose 360 mg/day CoQ10 for between 2 and 8 weeks has resulted in a fall in lactate concentration in the occipital cortex of patients. On withdrawal of CoQ10, lactate concentrations returned to their initial elevated level [75]. Although there may not have been a significant change in the behavioural and cognitive traits of these patients, this important finding paves the way for further research into the use of CoQ10 as a potential therapeutic agent in HD. There is evidence in the discussed literature of cellular benefits of CoQ10 supplementation, such as its effects on lactate concentrations. The challenge, however, is to reflect these findings in the phenotype. A lack of significant data could be attributed to the delivery of CoQ10 to its target tissue. Oral supplementation has to cross the blood–brain barrier (BBB) and as a result, the low doses trialed in the mentioned studies may not be sufficient to induce effects.

### 5.4. Friedrich’s Ataxia

FA is a rare autosomal recessive slowly progressive disease which results in progressive gait and limb ataxia, loss of position and vibration sense in the lower limbs as well as absence of deep tendon reflexes [78,79]. Diabetes mellitus, cardiac hypertrophy, optic atrophy and skeletal abnormalities may also be associated. Neuropathological changes begin in the dorsal root ganglia with a reduction in large neurons and loss of large myelinated fibres in the central axons and dorsal root nerves [80]. In 95% of patients, it is caused by an expansion of the guanine–adenine–adenine (GAA) repeat in intron 1 of the frataxin (FXN) gene on chromosome 9q21.11 [81,82]. This results in deficiency of frataxin, which is a mitochondrial matrix protein and is associated with iron metabolism and processing [83]. Post-mortem analysis of cardiac and skeletal muscle of FA patients showed a decrease in mitochondrial complexes I-III and aconitase activity, which are consequences of frataxin deficiency [84]. The role of frataxin was initially observed in yeast models with knockout of the homologue frataxin gene; mitochondrial iron accumulation, reduced levels of mitochondrial DNA and increased burden of oxidative stress were observed [85,86,87]. Hence, there is an accumulation of iron in the mitochondria and increased oxidative stress. Similarly, FA mice expressing reduced human-derived frataxin levels demonstrated progressive neurodegeneration and cardiac iron deposition as well as secondary demyelination and lipofuscin deposition with age, mainly due to oxidative stress at the biochemical level [88].

The role of CoQ10 as an antioxidant has been explored as a therapeutic strategy to slow the progress of FA. Lodi et al. investigated the combined treatment of 400 mg/day CoQ10 and 2100 IU/day Vitamin E for 6 months in a group of 10 FA patients with a control group and assessed neurological disease using the international cooperative ataxia rating scale (ICARS) as well as cardiac outcomes with echocardiography and magnetic resonance spectroscopy [89]. There were no benefits observed in ICARS or cardiac function at 3 months despite an increase in the cardiac phosphocreatine ratio [89]. At 4 years, the same group of patients showed no increase in the ICARS score, indicating disease stability, as well as a significant increase in fractional shortening in echocardiography (a measure related to the ejection fraction) [90]. In this group of patients, seven had better than expected neurological measures when compared to cross-sectional data from a larger group of FA patients and this led to the theory of CoQ10 responders and non-responders. This was further investigated in 50 patients who were randomly divided into high or low dose CoQ10 therapy combined with Vitamin E over a 2-year follow-up period [91]. Patients in the high-dose arm received 600 mg/day CoQ10 with 2100 IU/day vitamin E and those on low dose received 30 mg/day CoQ10 and 4 IU/day vitamin E. Both groups showed an increase in ICARS score and in 49% of those who completed the trial; ICARS scores were better than expected when compared to the cross-sectional group of FA patients [91]. Post-hoc analysis indicated that lower baseline-serum CoQ10 levels may be a predictor to response to CoQ10 therapy since the responder group had significantly lower baseline serum Co10 levels [91]. We can see that although CoQ10 supplementation alone yields little results in neurodegenerative diseases, combination therapy with vitamin E has been more promising. There is yet scope for further research with vitamin E combination in the management of AD and PD.

## 6. Conclusions

CoQ10 is a ubiquitous enzyme with antioxidant properties and functions integral to ATP production by acting as a cofactor to support enzymes in the cell. CoQ10 deficiency presents phenotypically in the most metabolically active tissues such as skeletal muscle, brain and the retina. Ageing is associated with a decline in CoQ10 and increased oxidative stress. Retinal diseases and neurodegenerative disorders such as AD, PD, HD and FA have an increased burden of oxidative stress, which cannot be counteracted by the reduced CoQ10 levels observed in these disease states. Although animal models have shown promising results in the role of CoQ10 supplementation in these conditions, introducing this to humans poses several challenges, in particular with regards to delivering CoQ10 in a safe manner to sites of disease burden. Furthermore, despite studies showing improvements in oxidative markers, it remains unclear whether this translates to observable clinical benefits. Moreover, limitations lie in the bioavailability of treatments accessing the CNS through oral administration and the retina through topical medication. From the existing literature, it can be noted that in retinal disease, high-dose CoQ10 supplementation has beneficial effects, whereas in neurodegenerative conditions, positive effects are more difficult to see. AD, in particular, does not yield significant differences with CoQ10 supplementation. This could be because topical therapy is delivered to the target tissue. However, oral supplementation has reduced efficacy. Man-made alternatives to CoQ10 such as idebenone have shown promising results in neurodegenerative disorders such as AD; however, more evidence is needed on the effectiveness of such supplements. Further studies need to be conducted to delineate the mechanism of ageing, CoQ10 and disease pathogenesis.

## Abbreviations

MDPI	Multidisciplinary Digital Publishing Institute
DOAJ	Directory of open access journals
TLA	Three letter acronym
LD	Linear dichroism
